# All Solid-State Poly (Vinyl Chloride) Membrane Potentiometric Sensor Integrated with Nano-Beads Imprinted Polymers for Sensitive and Rapid Detection of Bispyribac Herbicide as Organic Pollutant

**DOI:** 10.3390/molecules24040712

**Published:** 2019-02-16

**Authors:** Nashwa S. Abdalla, Maha A. Youssef, H. Algarni, Nasser S. Awwad, Ayman H. Kamel

**Affiliations:** 1 Chemistry Department, Faculty of Science, Ain Shams University, P.O. 11566 Cairo, Egypt; anoooosh311@gmail.com; 2 Analytical Chemistry and Control Department, Hot Laboratories Center, Atomic Energy Authority of Egypt, P.O. 13759 Abu Zaabal, Cairo, Egypt; maha_hasa@yahoo.com; 3 Department of Physics, Faculty of Science, King Khalid University, P.O. Box 9004 Abha, Saudi Arabia; halgarni@kku.edu.sa; 4 Department of Chemistry, Faculty of Science, King Khalid University, P.O. Box 9004 Abha, Saudi Arabia; nsawwad20@yahoo.com

**Keywords:** bispyribac sodium, solid-contact ISEs, screen-printed, molecularly imprinted polymers, organic pollutant

## Abstract

All-solid-state potentiometric sensors were prepared by using polyaniline (PANI) as the solid contact material. A film of PANI (thickness approximately being 0.25 µm) was deposited on a solid substrate (carbon screen printed platform). The PANI layer was subsequently coated with an ion-selective membrane (ISM) containing uniform-sized molecularly imprinted nanoparticles to produce a solid-contact ion-selective electrode (SC/ISE) for bispyribac herbicide (sensor I). In addition, aliquat 336 was also used as an ion exchanger in plasticized PVC membrane (sensor II). The proposed sensors revealed a remarkably improved sensitivity towards bispyribac ions with anionic slopes of −47.8 ± 1.1 (r^2^ = 0.9995) and −44.4 ± 1.4 (r^2^ = 0.9997) mV/decade over a linear range 1.0 × 10^−2^–8.6 × 10^−6^ M, 1.0 × 10^−2^–9.0 × 10^−6^ M and detection limits of 1.33 and 1.81 µg/mL for sensors I and II, respectively.Selectivity of both sensors is significantly high for different common pesticides and inorganic anions. The potential stability of the SC/ISEs was studied using chronopotentiometry. Electrochemical impedance spectrometry was used to understand the charge-transfer mechanisms of the different types of ion-selective electrodes studied. The impedance response of the electrodes was modelled by using equivalent electrical circuits. The sensors were used for a direct measurement of the bispyribac content in commercial herbicide formulations and soil samples collected from agricultural lands planted with rice and sprayed with bispyribac herbicide. The results agree fairly well with data obtained using HPLC method.

## 1. Introduction

Herbicides are commonly used in agriculture to control the growth of unwanted weeds. They act as plant-hormone mimics which can control the cell division, bud generation, root initiation, etc. [1]. To get rid of rice weed, bispyribac-sodium (BS) is the most commonly recommended herbicide with a broad spectrum and large effect. It has an inhibition effect on the activity of acetolactate synthetase enzyme that affects on plant growth. Therefore, bispyribac has been used for controlling a broad range of weeds [2,3]. It clearly showed a low risk to wild mammals, birds, earthworms, bees, aquatic invertebrates and fish, but excessive use may lead to adverse effects on human health, wildlife and the environment [4]. Its toxicology reports have not yet been reviewed by the EPA and toxic endpoints have not yet been established, specifically chronic and acute oral toxicity endpoints. A series of standards for estimating the maximum residue limits (MARLs) of (BS) have been developed by many countries or regions. For Japan and USA demands, the (MARLs) should be in rice ≤0.1 and ≤0.02 mg/kg, respectively. For EU demands, it should be ≤0.01 and ≤0.02 mg/kg in agricultural products and rice, respectively [5]. For surface water, the maximum levels permitted are 1 μg/L for individual pesticides and 5 μg/L for the total pesticides [6]. In Egyptian and American legislations, they established maximum limits for pesticides in drinking and environmental water, but bispyribac-sodium is not included.

Few analytical methods were developed and cited in the literature to estimate (BS) in different matrices. Some of these methods are HPLC [7,8,9,10,11] and LC coupled with mass spectrometry [12,13]. Although some of these methods are sensitive and selective, they are also time-consuming, offer complicated operation steps, require an expensive instrumental set up and sample pretreatments. In addition, the obtained results have not been feasible, with problems experienced including low extraction ratios and clean-up conditions [10,14]. Therefore, it is important to improve a simple, easy and cheap method for the detection of pesticides.

In recent years, potentiometric ion sensors, or the so called “ion-selective electrodes (ISEs)” have become an attractive technique for trace-level environmental assessment. This can be attributed to their intrinsic advantages such as excellent selectivity, ease of use and high reliability [15,16,17,18]. However, it should be noted that most of these potentiometric sensors were introduced for inorganic ions determination, but potentiometric sensors for organic anions have not been widely reported. Nowadays, potentiometric sensors integrated with molecularly imprinted polymer (MIP) exhibit a great potential to significantly change this situation [19,20,21,22]. During the last few years, potentiometric sensors integrated with MIPs showed success in organics detection. Almost all of these developed ISEs were in traditional liquid contact mode in which lower detection limits have been restricted by zero-current trans-membrane ion fluxes [23]. This has serious limitations for their use in environmental trace-level analysis. Solid-contact potentiometric ISEs can eliminate the inner filling solution. They are characterized by their convenient storage and maintenance, ease of miniaturization, and lower detection limit because of diminished ion fluxes [24,25]. As one of solid-contact electrodes, screen-printed electrodes have shown to be a good device for sensor miniaturization, and they are simple, cheap and easy to be fabricated for mass production. These good features of screen-printed electrodes make this type of electrodes promising for the detection of organic species. So far, only one research finding about screen-printed potentiometric sensors based on MIPs have been reported in literature [21].

In this paper, we present for the first time a novel screen-printed potentiometric sensor integrated with MIPs for detecting bispyribac herbicide. Polyaniline (PANI) film was used as the solid contact. The sensing element incorporated into the polymeric membrane was MIP nano-beads, which were employed as the receptors for the selective recognition of bispyribac. The proposed sensors could offer a high sensitivity and selectivity for potentiometric detection of bispyribac in commercial formulations and soil samples.

## 2. Results and Discussions

### 2.1. Characterization of MIP Particles

**Figure 1:**
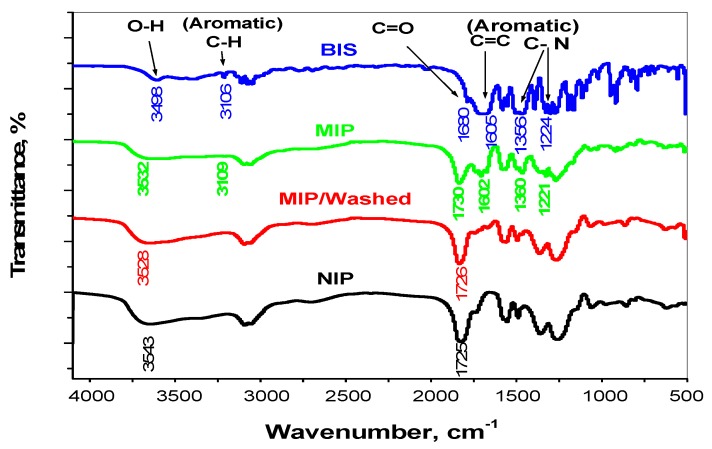
FT-IR spectra for bispyribac (BIS), bispyribac/MIP, MIP/washed and NIP beads.

As shown in Figure 1, the FTIR spectra of bispyribac/MIP, washed/MIP, NIP particles and bispyribac were characterized. A υ_O–H_ stretch vibration peak in bispyribac appeared at 3498 cm^−1^ due to the presence of a carboxylic group (COOH). This characteristic peak appeared at 3532 cm^−1^ in bispyribac/MIP. The shift is attributed to hydrogen bond formation between (COO^−^) in bispyribac molecule and (H^+^) in methacrylic acid monomer during the polymerization process. For washed/MIP, a O–H peak appeared at 3547 cm^−1^ which is very close to the O–H peak appeared in NIP beads (3543 cm^−1^). For the υ_O-H_ bending vibration peaks, they appeared at 1449 and 1394 cm^−1^ in bispyribac and shifted to 1458 and 1392 cm^−1^ in bispyribac/MIP. In both NIP and washed/MIP, these peaks appeared in low intensities at 1455 and 1398 cm^−1^, respectively. Aromatic C–H stretch peaks (signed to an aromatic ring in bispyribac) appeared at 3106 cm^−1^ in bispyribac while it was shifted to 3109 cm^−1^ in bispyribac/MIP. This peak is not present in both NIP and washed/MIP particles. In addition, aromatic C–H “oop” peaks appeared in 916 and 645 cm^−1^ in bispyribac were shifted to 921 and 646 cm^−1^ in bispyribac/MIP and not present in both NIP and washed/MIP particles. This confirms the complete removal of bispyribac from the MIP beads after washing. Aliphatic C–H stretch vibration peaks (originated from methyl group) appeared at 2968 and 2943 cm^−1^ in bispyribac while they were shifted to 2989 and 2955 cm^−1^ in bispyribac/MIP, respectively. In both NIP and washed/MIP, these peaks appeared at 2988 and 2956 cm^−1^ which confirms the absence of bispyribac molecule. For the carbonyl groupυ_c=o_ stretch appeared at1730 cm^−1^ in bispyribac/MIP and shifted to 1725 cm^−1^ in both NIP and washed/MIP. For aromatic C=C stretch vibration peaks (signed to an aromatic ring), they were noticed at 1605 and 1585 cm^−1^ in bispyribac and shifted little to 1602 and 1572 cm^−1^ in bispyribac/MIP. Theses peaks completely disappeared in both NIP and washed/MIP due to the absence of bispyribac molecule. Aromatic C–N stretch vibration peaks (signed to an aromatic ring) appeared at 1356, 1224, 1196 cm^−1^ in bispyribac and shifted to 1360, 1221, 1192 cm^−1^ in bispyribac/MIP while they disappeared in NIP and washed/MIP. From all of the above, the imprinting process of bispyribac using MAA as a functional monomer is succeeded and complete removal of the template from the backbone of the imprinted polymer is achieved.

**Figure 2:**
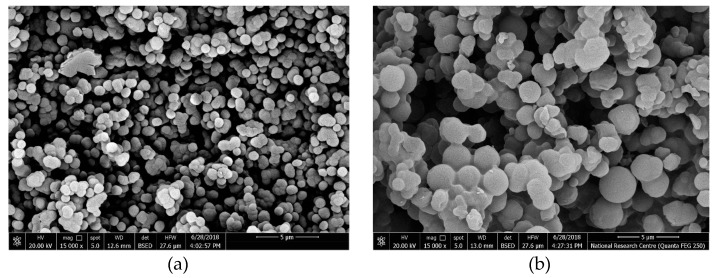
SEM images of (**a**) MIP and (**b**) NIP nanobeads.

To examine the morphologies of the obtained bispyribac MIP particles, scanning electron microscopy (SEM) was used. As shown in Figure 2a, the bispyribac imprinted particles were of uniform-size and spherical with a diameter distribution of 100–400 nm. These uniform-sized nano-beads give a good dispersion in the polymeric ISE membrane. This could reduce the membrane resistance and induce more binding sites available in the membrane [26]. The preparation of NIP nano-beads using the same protocol showed a similar morphological structure and particle size distribution as in MIP particles (Figure 2b).

**Figure 3:**
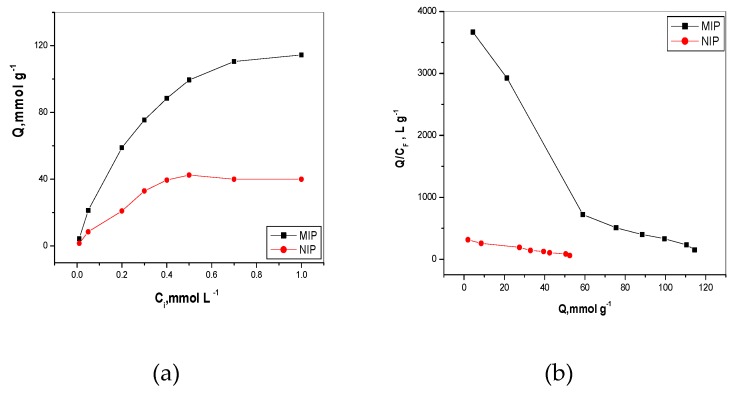
(**a**) Binding isotherm (*Q* = mmol/g) is plotted as a function of byspyribac concentration, *C* = mmol/L); and (**b**) Scatchard plot (*Q/C free* = L/g is plotted as a function of *Q* = mmol/g). *Q* is the amount of byspyribac bond per g of polymer; t = 25 °C; V = 10.0 mL; binding time: 20 h.

The binding capacity (*Q*) of bispyribac-MIP or NIP was calculated using the following equation:

(1) $$Q=\frac{\left(Bounded\;BS\right)}{\left(MIP,NIP\right)\left(g\right)}=\frac{\left(C_{1}-C_{2}\right)\times V}{\left(MIP,NIP\right)\left(g\right)}$$

where *C*_1_ is the initial bispyribac concentration (mM), *C*_2_ is the final bispyribac concentration (mM), *V* is the volume of the solution (mL) and *g* is the mass of adsorbent polymer. As shown in Figure 3a, the adsorption capacity was obviously increased by increasing the initial concentration until reaching equilibrium saturation above 0.7 and 0.5 mM for both MIP and NIP, respectively. Also, at the same initial concentration, the adsorption capacity of MIP clearly appeared to be higher than that of NIP. This refers to the presence of specific recognition sites formed in MIP which can bind bispyribac selectively. The data obtained from Figure 3a was used to precede Scatchard analysis for calculating the apparent maximum adsorption capacity (*Q_max_*, mM/g) and dissociation constant at the binding site (*K_d_*). As shown in Figure 3b, there are two segments in the curve corresponding to low and high concentration range of bispyribac/g MIP beads. This indicated that the binding sites of MIP were non-uniform and the affinities of the binding sites are heterogeneous. In contrast, one linear line segment of NIP beads appeared, indicating that the affinities of the binding sites were homogenous. The two linear regression equations of the two segments of MIP are: *Q/C* = −0.0547*Q_max_* + 3.9826 (r^2^ = 0.9979) and *Q/C* = −0.0086*Q_max_* + 1.1650 (r^2^ = 0.9883) and the linear regression equation of the segment of NIP is *Q/C* = −0.0047*Q_max_* + 0.3097 (r^2^ = 0.9924). The values of *K_d_* and *Q_max_* of high-affinity sites of MIP were 18.27 µM and 73.08 µmol/g, respectively. In low-affinity sites, the values were 115.98 µM and 135.23 µmol/g, respectively. In contrast, *K_d_* and *Q_max_* values of NIP beads were 221.59 µM and 65.5 µmol/g. This can indicate that a *Q_max_* value of NIP beads is lower than of high and low-affinity sites of MIP beads.

From all of the above, we can conclude that the binding capacity of MIP toward bispyribac was higher than that of the binding capacity in NIP. This is attributed to the presence of specific binding cavities in MIP beads towards bispyribac, which are formed during the polymerization process. In NIP beads, bispyribac can only be bounded on the surface of the NIP polymer.

### 2.2. Characteristics of the Proposed Sensors

**Figure 4:**
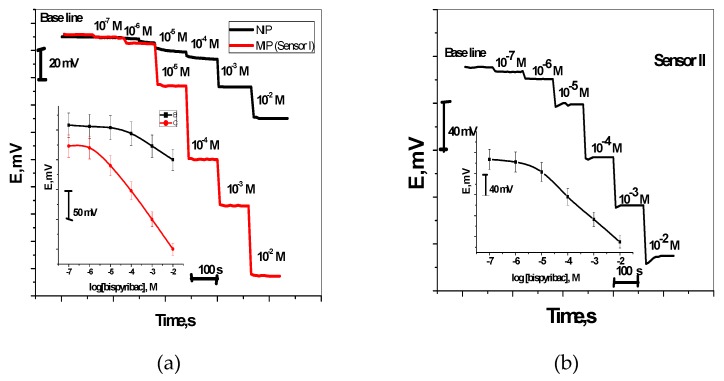
Potentiometric response curves obtained screen-printed ISEs integrated with MIP nanobeads curve (**a**) and aliquat curve (**b**).

Bispyribac Sodium selective membranes have two electroactive materials. These include MIP as anionic receptor (sensor I) and aliquate 336 as an anion exchanger (sensor II). The potential signals measured by these sensors were presented in Figure 4. For sensor I, it exhibited a sub-Nernstian response towards bispyribac ions with anionic slopes of −47.8 ± 1.1 (r^2^ = 0.9995) mV/decade over a linear range 1.0 × 10^−2^–8.6 × 10^−6^ M with a detection limit 1.33 µg/mL. For bispyribac sensor based on aliquate336 (sensor II), it exhibited a potentiometric response towards bispyribac ions with sub-Nernstian slopes of −44.4 ± 1.4 (r^2^ = 0.9997) mV/decade over the linear concentration range 1.0 × 10^−2^–9.0 × 10^−6^ M with detection limits of 1.81 µg/mL.

As a control, membrane sensors based on NIP nano-beads were tested. These sensors showed a worse response performance towards bispyribac than compared with the response of the MIP nano-bead-based sensors for all measuring concentrations under the same conditions (Figure 4a). They exhibited anionic slopes of −22.2 ± 1.3 (r^2^ = 0.9986) mV/decade over a linear range 1.0 × 10^−2^–1.0 × 10^−4^ M with a detection limit 4.5 × 10^−5^ M. This can confirm that the potential response obtained by these sensors is induced by the specific recognition interactions between the MIP binding sites in the membrane and bispyribac.

The capability of the screen-printed electrodes to resist long-term storage was checked. Pre-conditioning for the sensors for 1 h in 10^−5^ M bispyribac then in 10^−2^ M NaCl for 2 h is firstly done. Preliminary tests were carried out by using the sensors, storing them in a dry place, and retesting their performance after a given time. After re-calibrating these sensors every day over a week, no significant changes in the analytical performance were noticed. This revealed that the sensors can be used, dried and stored. Repeating the calibration of the sensors for longer periods of time, and reusing periods of at least four weeks have been tested. It was noticed that after 10 days of daily use, the limit of detection increase up to 10^−5^ M, and the potentiometric response declined and the sensitivity decrease. Regardless of this issue, considering the disposable nature of these sensors, long-life span is not considered a big problem.

The dynamic response of these sensors was tested over the concentration range 10^−7^–10^−2^ M. As shown in Figure 4a,b, the proposed sensors were insensitive to the change of bispyribac level when concentration was below 5 × 10^−6^ M. But a stable performance was observed at high concentrations (e.g., above 10^−5^ M).

**Figure 5:**
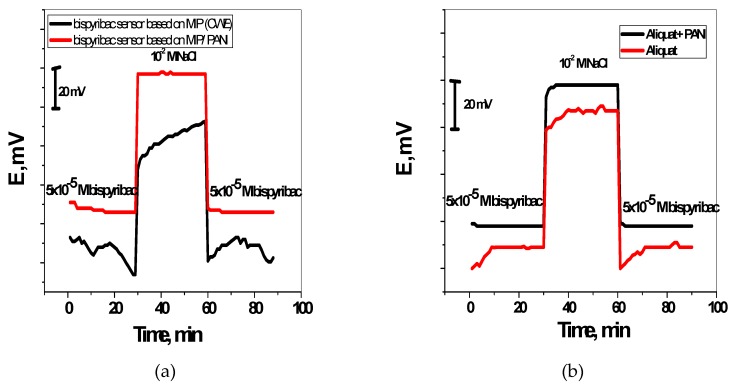
Water-layer tests for the bispyribac-ISE with and without PANI as the solid contact using MIP nano-beads (**a**) and aliquat (**b**) membrane based sensors.

To examine the water-layer formation between the membrane and the PANI layer, the potentiometric water layer test was carried out. Figure 5 illustrated the potentiometric response of the screen-printed electrodes in presence and absence of PANI, which were initially measured in 5 × 10^−5^ M bispyribac-sodium, then in 10^−2^ M NaCl and again in 5 × 10^−5^ M bispyribac-sodium solutions. Clearly, a water layer was formed in the screen-printed electrodes without PANI layer. This can be seen from the obvious potential drift when it was changed back from the interfering ion (Cl^−^) to the bispyribac ion. By contrast, the electrodes modified with PANI layer exhibited a stable behavior and the equilibrium was reached rapidly after the alteration. This presents evidence for the hydrophobicity of PANI layer through no water layer formation.

### 2.3. Potential Stability

**Figure 6:**
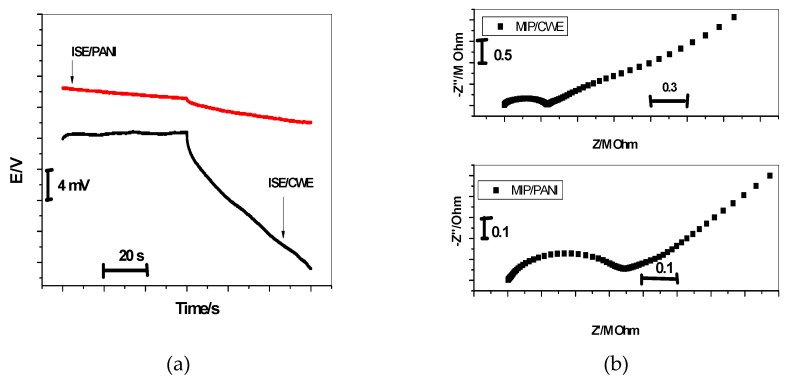
Chronopotentiometry (**a**) and Impedance plot (**b**) for bispyribac/MIP-ISEs with and without PANI as a solid contact material.

Constant-current chronopotentiometry has been developed by Bobacka’s group and widely used to evaluate the short-term potential stability of the all-solid-state ISEs [27]. Currents of ±1 nA were applied on the MIP/CWE and MIP/PANI electrodes and the potential responses were measured in 10^−2^ M bispyribac solution. From the chronopotentiogram shown in Figure 6a, the total resistance (*R_tot_*) of the MIP/CWE and MIP/PANI electrodes is estimated to be about 1.1 and 0.5 MΩ, respectively. For MIP/PANI electrode, the potential stability is 36.9 ± 3.2 μV/s (*n* = 3), which is much lower than that of the MIP/CWE electrode under the same conditions (∆*E*/∆*t* = 223.1 ± 11.2 μV/s (*n* = 3). Due to the high double layer capacitance of PANI, the presence of PANI as an ion-to-electron transducer would facilitate faster charge transport between interfaces and offer more stable potential responses for SC-ISEs.

Furthermore, the potential drift (∆*E*/∆*t*) of the all-solid-state bispyribac-ISEs can be related to the low-frequency capacitance (*C*_L_) of the solid contact and the current (*i*) as follows:

Potential drift = Δ*E*/Δ*t* = *i*/*C*_L_ (2)

The capacitances of MIP/PANI based SC-ISE and CWE were calculated to be 27.1 ± 0.4 µF and 4.48 ± 0.6 µF, respectively. From the above results, we can conclude that there is a clear relationship between the potential stability (∆*E*/∆*t*) or the capacitance (*C*_L_) and the presence of PANI layer in the solid contact.

### 2.4. Electrochemical Impedance Spectrometry

All the electrochemical impedance measurements were performed on the proposed sensors in a 0.01 M bispyribac solution using autolabpotentiostat/galvanostat (Metrohom). Double junction Ag/AgCl was used as a reference electrode, and a platinum plate was used as the auxiliary electrode. The impedance measurements covered the frequency range from 10 kHz to 0.1 Hz and the amplitude of the sinusoidal excitation signal was 100 mV. As shown in Figure 6b, the impedance plot for all-solid-state bispyribac ISEs shows that a high-frequency semicircle can be related to the bulk impedance of the membrane (*R*_b_ = 0.35 ± 0.03 MΩ) in parallel with its geometric capacitance (*C*_g_) [28]. For the MIP/CWE, the bulk resistance obtained by EIS (*R*_b_ = 0.35 ± 0.03 MΩ) is in good agreement with the chronopotentiometric results (*R* = 0.5 ± 0.02 MΩ). The geometric capacitance is *C*_g_ = 0.32 ± 0.02 nF. For the low-frequency branch (semicircle), it is very characteristic for the type of solid contact used [29]. The CWE shows a large low-frequency semicircle. This can be related to the small double-layer capacitance and the large charge-transfer resistance at the “blocked” interface between carbon in the screen-printed platform and the ion selective membrane (ISM). This is possibly connected with the long-term potential drift observed for the CWEs. For MIP/PANI-ISEs, a depressed low-frequency semicircle is obtained due to the charge transfer across the PANI layer. The low-frequency capacitance (*C*_L_) for CWE and MIP/PANI-ISE were *C*_L_ = 3.12 ± 0.17 µF and *C*_L_ =25.3 ± 1.1 µF, respectively.

### 2.5. Selectivity

The selectivity values of the proposed sensors were performed and calculated using modified separate solution method reported by Bakker et al. [30]. The logarithmic selectivity coefficients for bispyribac (log$K_{M,X}^{pot}$) over other inorganic anions and similar structure analogs were summarized in Table 1. Evidently, the proposed sensors exhibited excellent selectivity towards bispyribac over other pesticides such as diquate, acetamiprid, dinotefuran, imidachloprid, cyromazine, flucarbazone and some common inorganic anions.

From all of the above, the results obtained reflect excellent selectivity for the proposed sensors, and offer a great potential for trace-level monitoring of bispyribac in environmental samples as a low cost, disposable alternative for these applications where conventional sensors are not affordable.

### 2.6. Analytical Applications and Sample Analysis

The proposed sensors were applied for Bispyribac-sodium determination in Nominee-kz, 3% soluble liquid (SL) formulation in triplicate using the standard addition method. The results are shown in Table 2. These data were compared with results obtained by measuring Bispyribac using HPLC [31].

Application to soil samples collected from agricultural lands planted with rice and sprayed with bispyribac herbicide was carried out. Three different soil samples were collected after one day of spraying bispyribac herbicide to the land. Before measurements, the sensors were firstly calibrated by using the linear equation for bispyribac. Then, the same batch of sensors can be used in the sample analysis directly. Similar results are obtained using HPLC method (Table 3).

It can be observed that the proposed MIP-based sensor have promising feasibility for the determination of bispyribac herbicide in complex samples.

## 3. Materials and Methods

### 3.1. Chemicals and Reagents

Bispyribac sodium (99%), Diquate dibromide (98.3%), Dimethoate (98%) and Cyromazine (98.5%) were purchased from Dr. Ehrenstorfer GmbH (Germany). Methomyl (98.5%) and Oxamyl (99%) were purchased from Fluka (Ronkonkama, NY). Polyaniline (emeraldine salt) (average M_w_ > 15,000, 3–100 μm particle size), methacrylic acid (MAA), ethylene glycol dimethacrylate (EGDMA), acetonitrile and benzoyl peroxide (BPO) were purchased from Sigma-Aldrich Inc. (St. Louis, MO, USA). Aliquat 336 was purchased from Acros (Spain). High molecular weight poly (vinylchloride) (PVC), dioctyl phthalate (DOP) was obtained from Fluka AG (Buchs, Switzerland). Tetrahydrofuran (THF) was freshly distilled prior to use. For pesticide technical formulation, a Nominee-kz, 3% soluble liquid (SL) of bispyribac sodium, was purchased from Kafr El-Zayat Pesticides& Chemicals Company (Gharbia, Egypt). All other chemical reagents were of analytical grade and used without any further purification.

A stock bispyribac sodium solution (10^−2^ M) was prepared by dissolving 0.45 g pure bispyribac sodium in 100 mL distilled water. Working solutions (10^−2^–10^−6^ M) were prepared by accurate dilutions and stored in brown bottles.

### 3.2. MIPs Synthesis

**Figure 7:**
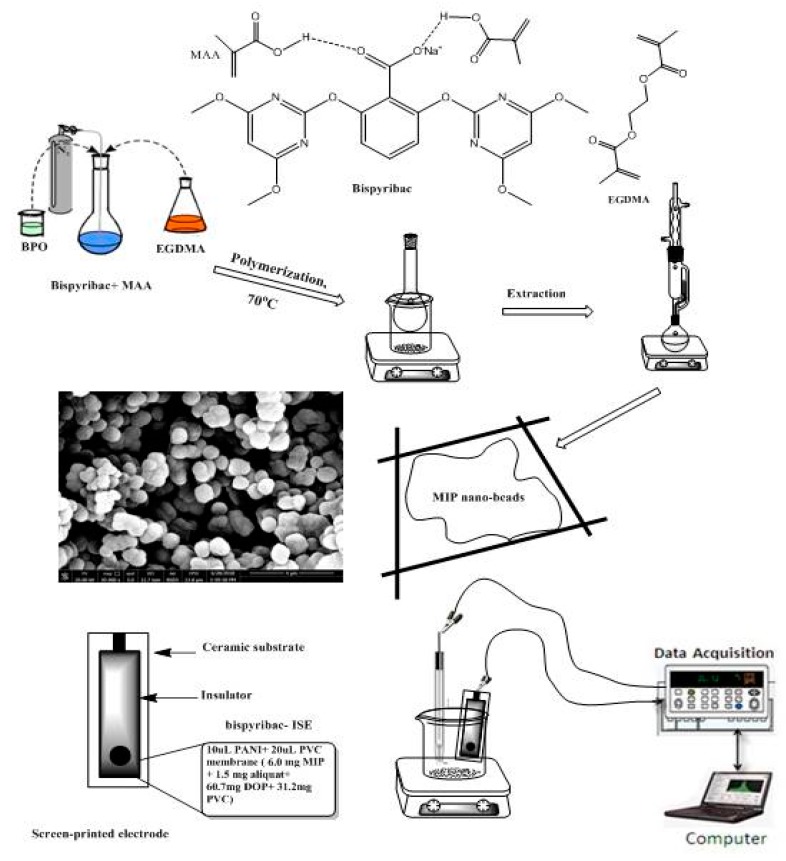
Schematic representation for MIPs synthesis and screen- printed electrode.

The precipitation method was used for bispyribac MIP particles synthesis as shown in Figure 7. In brief, the template bispyribac (1.0 mmol), methacrylic acid (MAA, 3.0 mmol), ethylene glycol dimethacrylate (EGDMA, 3.0 mmol), and free radical initiator benzoyl peroxide (BPO, 60 mg) were dissolved in a 25 mL glass-capped bottles in acetonitrile (15 mL), then the mixture was sonicated for 10 min for homogeneity. The solution was purged with N_2_ for another 10 min to expel the dissolved oxygen. The temperature was set at 75 °C for 18 h for complete polymerization in oil bath. After polymerization, the template was removed by soxhlet extraction using methanol/acetic acid (8/2, *v*/*v*) and methanol until no absorption of bispyribac was observed with a Shimadzu UV/VIS spectrophotometer (Model UV-1601, Shimadzu, Japan). The resulting polymer was left to dry at an ambient temperature for 24 h. Non-imprinted polymer (NIP) was prepared under identical conditions without the template.

### 3.3. Sensors Preparation and EMF Measurements

As shown in Figure 7, the conductive carbon layer present in the screen-printed sensors, a 10 μL of 3 mg/mL PANI (dissolved in THF) was applied by drop casting on this layer. After drying the applied conducting polymer, a layer with a thickness close to 0.25 µm was formed and used as the solid contact layer. The membrane cocktail used for fabricating the screen-printed carbon sensors was prepared by dissolving 100 mg of components in 1.0 mL THF: MIP or NIP (6.0 wt%), PVC (32.2 wt%), and DOP (61.8 wt%) and aliquat 336 (2.5 wt%), PVC (33.2 wt%), and DOP (64.3 wt%) for sensors I and II, respectively. 20 μL of the membrane cocktail was drop-cast onto the screen-printed carbon electrode and allowed to dry for 6 h. Before the measurement, the sensors were conditioned for 2 h in 10^−3^ M of bispyribac solution.

All measurements of EMF were performed at ambient temperature using Orion pH/mV meter (Model SA 720, Cambridge, MA, USA). An Orion Ag/AgCl double junction reference electrode (type 90-02, USA), filled with 10% (*m*/*v*) KNO_3_ solution was used to complete the electrochemical cell. The EMF values were corrected for the liquid-junction potential according to the Henderson equation. The ion activity coefficient was calculated from the modified Debye-Hükel equation [32].

### 3.4. Adsorption Isotherm

Adsorption isotherms were carried out to calculate the binding capacity (*Q*) which represents the maximum amount of bispyribac adsorbed per gram of MIP or NIP particles. 20-mg of either MIP or NIP particles were placed in contact with 10-mL of different bispyribac concentrations (0.05 to 1.0 mM) and left together at room temperature under static equilibrium. After 12 h, the mixture was separated by centrifugation (4000 rpm, 10 min.) and then filtered through a 0.45-μm filter. Free bispyribac concentrations in the supernatant were measured by UV-spectrophotometry at 246 nm. The amount of bispyribac bound to the polymers was calculated by subtracting the concentration of free bispyribac from the initial bispyribac concentration.

### 3.5. Applications to Real Samples

To test the applicability of the proposed sensors, they were introduced to assess bispyribac in commercial soluble liquid (SL) formulation, soil and agricultural waste water samples collected from different agricultural lands planted with rice and sprayed with bispyribac herbicide.

A locally present herbicide (Nominee-kz, 3% soluble liquid (SL) of bispyribac sodium) was purchased from Kafr El-Zayat Pesticides& Chemicals Company (Gharbia, Egypt). Accurately 0.5–1.0 mL of the formulation were transferred to 100-mL measuring flask and diluted to the mark. The test solutions were measured and the potential reading was compared with a calibration plot prepared from (10^−2^ to 10^−6^ M) standard bispyribac solutions under similar conditions. For soil analysis, 250 g soil from agricultural lands planted with rice and sprayed with bispyribac herbicide was collected and soaked into 250 mL water for 1 h. After filtration, the obtained filtrate was then spiked with various concentrations of bispyribac and analyzed by the proposed sensor using the standard addition method.

## 4. Conclusions

A polyaniline (PANI)-based screen-printed potentiometric sensor for the determination of bispyribac was fabricated and characterized. This study reinforced the excellent characteristic of PANI as a good solid contact for solid contact-ISEs. The PANI intermediate layer can greatly enhance the electron-transfer transduction and eliminate the water layer formation between the solid contact layer and the membrane sensor. Integration of molecularly imprinted polymer (MIP) receptors with screen-printed potentiometric sensors have been successfully fabricated for detection of organic species. The sensors revealed rapid and reasonable sub-Nernstian slope of −47.8 ± 1.1 and −44.1 ± 1.4 mV/decade together with a low detection limits 1.33 and 1.81 µg/mL for sensors I and II, respectively. They offered the advantages of fast response, reasonable selectivity, good accuracy, possible interfacing with computerized and automated systems, and a satisfactory accuracy for real analysis.

## Figures and Tables

**Table 1:** Potentiometric selectivity coefficients, log$K_{M,X}^{pot}$, of the proposed screen-printed ISEs.

Interfering Ion	* Log *K^pot^_x,y_*	
	Sensor I	Sensor II
Diquate	−5.05 ± 0.2	−5.04 ± 0.5
Acetampirid	−4.73 ± 0.4	−4.63 ± 0.2
Dinotefuran	−5.17 ± 0.5	−3.12 ± 0.3
Imidachloprid	−5.32 ± 0.3	−5.98 ± 0.8
Cyromazine	−5.32 ± 0.7	−5.35 ± 0.1
Flucarbazone	−2.51 ± 0.1	−0.24 ± 0.05
Cl^−^	−5.05 ± 0.4	−4.75 ± 0.5
Br^−^	−5.15 ± 0.2	−2.97 ± 0.4
SO_4_^2−^	−5.57 ± 0.3	−5.08 ± 0.3
CH_3_COO^−^	−3.96 ± 0.2	−4.21 ± 0.1
NO_3_^−^	−4.25 ± 0.6	−3.06 ± 0.2

* Mean value obtained from three corresponding pairs of concentrations of bispyribac ion and the respective interfering anion in the Nernstian response range ± standard deviation.

**Table 2:** Potentiomeric determination of bispyribac in commercial herbicide formulation using MIP/PANI membrane based sensor.

Commercial Product	Label (*w*/*v* %)	^a^ Found			
		Potentiometry	RSD, %	HPLC [28]	RSD, %
Nomenee-kz, Kafr El-Zayat Pesticides& Chemicals Company (Gharbia, Egypt)	3	2.97 ± 0.02	99.0	2.99 ± 0.05	99.6

^a^ Average of five measurements ± standard deviation.

**Table 3:** Determination of bispyribac in some soil samples were collected after one day of spraying bispyribac herbicide to the land.

Sample	Amount of Bispyribac (µg/g)	
	Potentiometry	HPLC [28] ^a^
Sample 1	8.8 ± 0.9	9.2 ± 0.2
Sample 2	10.4 ± 0.4	9.7 ± 0.1
Sample 3	14.3 ± 0.7	13.1 ± 0.3

^a^ Average of five measurements ± standard deviation.
